# Treatment burden for patients with multimorbidity: cross-sectional study with exploration of a single-item measure

**DOI:** 10.3399/BJGP.2020.0883

**Published:** 2021-04-20

**Authors:** James E Morris, Paul J Roderick, Scott Harris, Guiqing Yao, Sam Crowe, David Phillips, Polly Duncan, Simon DS Fraser

**Affiliations:** School of Primary Care, Population Sciences and Medical Education, Faculty of Medicine, University of Southampton, Southampton General Hospital, Southampton.; School of Primary Care, Population Sciences and Medical Education, Faculty of Medicine, University of Southampton, Southampton General Hospital, Southampton.; School of Primary Care, Population Sciences and Medical Education, Faculty of Medicine, University of Southampton, Southampton General Hospital, Southampton.; Department of Health Sciences, University of Leicester, Leicester.; Public Health Dorset, Dorchester; Public Health Dorset, Dorchester.; Centre for Academic Primary Care, University of Bristol, Bristol.; School of Primary Care, Population Sciences and Medical Education, Faculty of Medicine, University of Southampton, Southampton General Hospital, Southampton.

**Keywords:** cross-sectional studies, general practice, multimorbidity, treatment burden

## Abstract

**Background:**

Treatment burden is the effort required of patients to look after their health, and the impact this has on their wellbeing. Quantitative data on treatment burden for patients with multimorbidity are sparse, and no single-item treatment burden measure exists.

**Aim:**

To determine the extent of, and associations with, high treatment burden among older adults with multimorbidity, and to explore the performance of a novel single-item treatment burden measure.

**Design and setting:**

Cross-sectional postal survey via general practices in Dorset, UK.

**Method:**

Patients ≥55 years, living at home, with three or more long-term conditions (LTCs) were identified by practices. Treatment burden was measured using the Multimorbidity Treatment Burden Questionnaire. Data collected were sociodemographics, LTCs, medications, and characteristics including health literacy and financial resource. Associations with high treatment burden were investigated via logistic regression. Performance of a novel single-item measure of treatment burden was also evaluated.

**Results:**

A total of 835 responses were received (response rate 42%) across eight practices. Patients’ mean age was 75 years, 55% were female (*n* = 453), and 99% were white (*n* = 822). Notably, 39% of patients self-reported fewer than three LTCs (*n* = 325). Almost one-fifth (18%) of responders reported high treatment burden (*n* = 150); making lifestyle changes and arranging appointments were particular sources of difficulty. After adjustment, limited health literacy and financial difficulty displayed strong associations with high treatment burden; more LTCs and more prescribed regular medications were also independently associated. The single-item measure discriminated moderately between high and non-high burden with a sensitivity of 89%, but a specificity of 58%.

**Conclusion:**

High treatment burden was relatively common, underlining the importance of minimising avoidable burden. More vulnerable patients, with less capacity to manage, are at greater risk of being overburdened. Further development of a single-item treatment burden measure is required.

## INTRODUCTION

Treatment burden is the effort required of patients to look after their health and the impact this has on their functioning and wellbeing.^1^^–^^3^ Recognition of treatment burden in people living with long-term conditions (LTCs) is increasingly important, given the ageing populations of many countries, and increasing prevalence of LTCs and multimorbidity.^4^^,^^5^ The workload of health care can include ordering and taking medications, organising and attending healthcare appointments, monitoring health conditions, and modifying lifestyle behaviours.^6^ For people living with multimorbidity, clinicians may be following multiple single-disease guidelines, an important driver of polypharmacy and potentially of treatment burden.^7^^,^^8^ In the UK, the National Institute for Health and Care Excellence 2016 guidance for the assessment and management of multimorbidity^9^ advises taking an approach to care that includes *‘improving quality of life by reducing treatment burden’*.

Negative impacts of treatment burden are various and include those of both a psychological and practical nature, such as interference with daily activities, negative emotions, and strained relationships.^10^^,^^11^ Patients may seek to minimise disruptions not only through adaptation, but also by non-adherence to treatment.^10^^,^^12^ When thinking about treatment burden, it is important to consider patient ‘capacity’: the abilities, resources, and readiness to address the combined demands of treatment workload and daily life.^13^ Components of capacity may include socioeconomic resources such as financial wellbeing and support networks, health literacy, relevant knowledge and experience, and physical and mental functioning.^13^^,^^14^ When the workload exceeds available capacity, patients may be described not only as experiencing high treatment burden, but also as being overburdened — with the potential risk of involuntary non-adherence, disruption to care, and adverse health outcomes.^13^^,^^15^ A degree of treatment burden is perhaps inevitable in managing LTCs, but seeking to minimise unnecessary burden is important, as encapsulated by ‘minimally disruptive medicine’.^16^

Validated instruments to measure patient-perceived treatment burden are available.^17^^–^^21^ However, quantitative data on the extent of, and factors associated with, high treatment burden for patients with multimorbidity are limited. Measuring treatment burden is not currently part of routine care and existing measures are too long for time-constrained clinical encounters, particularly in UK general practice. The aims of this study were to determine the extent of treatment burden, and explore the characteristics associated with high treatment burden, among adults ≥55 years with three or more specified LTCs documented in their GP records; and to explore the performance of a novel single-item treatment burden measure in the same sample.

**Table:** How this fits in

Patients with multimorbidity may experience treatment burden from healthcare demands, but the extent of, and factors associated with, high treatment burden are not well understood. In this survey of older adults with multimorbidity, almost one-fifth reported ‘high’ treatment burden, which was associated not only with a greater number of long-term conditions and more prescribed regular medications, but also with characteristics indicating reduced ‘capacity’ to manage, namely limited health literacy and financial difficulty. GPs have a central role in incorporating these considerations into patient care, to try to ensure that patients are not overburdened. Additionally, existing measures of treatment burden are too time consuming for clinical use. A novel single-item measure of treatment burden was explored here, but performed moderately; further development of such a measure is therefore required.

**Box 1.** Inclusion and exclusion criteria for patients in this study

**Inclusion criteria**	**Exclusion criteria**
Aged ≥55 years Registered at a participating Dorset GP practice Diagnosed with ≥3 of the following long-term conditions, as recorded in the GP record (considered as separate conditions):^a^ Atrial fibrillation Coronary heart disease Heart failure Hypertension Peripheral arterial disease Stroke or transient ischaemic attack Diabetes Asthma Chronic obstructive pulmonary disease Depression Chronic kidney disease Epilepsy Osteoporosis Rheumatoid arthritis Parkinson’s disease Multiple sclerosis Inflammatory bowel disease Coeliac disease Osteoarthritis	Resident in a care home In receipt of palliative care Diagnosed with a severe mental health condition (psychosis, schizophrenia, bipolar disorder) Diagnosed with dementia Known to have active cancer (recorded within the last 3 years) Lacked mental capacity to participate Expressed wish not to participate in research Other reason, rendering a patient unsuitable to participate, at the discretion of a healthcare professional at the GP practice
Additionally: Only one person from a given household was eligible to participate Participating GP practices used the ‘SystmOne’ electronic medical record system^b^	

a Long-term conditions numbered 1–14 are Quality and Outcomes Framework (QOF) conditions; those numbered 15–19 are non-QOF conditions.

b SystmOne is the electronic GP record system predominantly used in practices throughout Dorset.

## METHOD

### Survey design and sample

A cross-sectional postal survey of older adults with multimorbidity was conducted in Dorset, England, between February and July 2019. Patients aged ≥55 years, living at home, and with three or more LTCs from a specified list, were identified for invitation from GP registers. Inclusion and exclusion criteria are shown in Box 1. Quality and Outcomes Framework clinical code clusters^22^ defined 14 LTCs, with Read codes^23^ (structured clinical terms) defining a further five. The 19 LTCs selected were common, readily identified from GP records, and represented a range of body systems. Conditions considered distinct regarding the likely avoidability, or impact on perception, of treatment burden (such as cancer or severe mental health diagnoses) were not included. The cut-off of ≥55 years defining ‘older’ adults was lower than a more orthodox threshold of ≥65 years because more deprived groups may experience multimorbidity at younger ages.^24^ Patients’ treatment burden was measured using the 10-item Multimorbidity Treatment Burden Questionnaire (MTBQ):^21^ a concise, simply worded instrument suitable for self-completion, with good coverage of burden domains and validated in a multimorbid population similar to that in the current study. These characteristics conferred greatest suitability relative to other candidate measures,^17^^–^^20^ which variously exhibited limitations such as wording complexity, narrowness of focus, or considerable length. Each MTBQ item is scored as follows: 0 (not difficult/does not apply), 1 (a little difficult), 2 (quite difficult), 3 (very difficult), and 4 (extremely difficult); for those completing five or more items, average item score is multiplied by 25 to yield a global score of 0–100. Treatment burden is categorised as none (global score 0), low (>0 and <10), medium (≥10 and <22), or high (≥22).

Self-reported data on a range of variables were collected through the survey, in areas including sociodemographics, prescribed regular medications, specific LTCs (corresponding to survey inclusion criteria), travel for health care, recent healthcare resource use, and health status/quality of life. Home ownership (dichotomised as homeowner/non-homeowner) served to measure socioeconomic status. Characteristics indicative of capacity were captured, including financial resource via the perceived level of difficulty in meeting the financial costs of health care (on a 5-point Likert scale from ‘no’ to ‘extreme’ difficulty), and health literacy via the Single Item Literacy Screener — which asks about the perceived frequency of needing help to read health-related written material (on a 5-point Likert scale, with descriptors ‘sometimes’, ‘often’, and ‘always’ indicating limited health literacy).^25^

A novel single-item measure of treatment burden was also explored: *‘On a scale of 0–10, where 0 is no effort and 10 is the highest effort you can imagine, how would you rate the amount of effort you have to put in to manage your health conditions?’*, with responders circling along a number-line. This measure did not undergo formal development; however, the wording and format embodied principles of existing measures of self-rated health (the EQ visual analogue scale)^26^ and pain (the numerical rating scale for pain).^27^ Additionally, MTBQ unidimensionality lent legitimacy to trialling a measure comprised of a single item.

Based on a 26.6% prevalence for high treatment burden from MTBQ validation data,^21^ identifying those with high burden would require at least 300 survey responses for a maximum 95% confidence interval (CI) width of +/–5%, considered sufficiently precise. nQuery (version 7.0) was used for the sample size calculation.

Survey documents were refined using a ‘think-aloud’ procedure (a cognitive-interviewing approach for pre-testing self-completion questionnaires),^28^ conducted with colleagues and a lay representative (see Supplementary Box S1 for details of survey questions and response options). This article reports on a subset of data and analyses.

### Recruitment, invitation, and response

Recruitment of GP practices sought eight geographically dispersed, socioeconomically diverse sites across Dorset. Each participating practice identified survey invitees by electronically searching their patient list with a supplied algorithm, and manually screening a random sample of resulting records — identifying at most 250 invitees meeting the inclusion/exclusion criteria. Reasons for manual exclusions were requested.

Practices posted survey packs to the invitees that comprised a personalised invitation letter, information sheet, consent form, survey booklet, and return envelopes. Freepost returns, GP practice endorsement, an uncomplicated means of participation, and a survey designed to minimise completion burden were used to maximise response rate, along with an online response platform offered via a weblink. Participants provided written (or online equivalent) consent; and could optionally consent to GP-record data sharing with the study team, and to receive a potential follow-up survey (both outwith the scope of this article).

### Statistical analyses

Survey data underwent manual database input, with a 5% double-entered sample facilitating error-rate estimation. SPSS Statistics (version 24) was used for analysis. Descriptive statistics were used to explore characteristics of the responder sample, non-response bias based on age and sex, and distribution of treatment burden. A binary outcome of high treatment burden versus no/low/medium burden combined was used (this dichotomy lending a focus to the most impacted group). Other variables acted as exposures. Univariable and multivariable logistic regression were used to identify associations with high treatment burden. Variables were considered for inclusion in a final multivariable model if deemed clinically important a priori or shown to be statistically significant (at *P*<0.05) at the univariable stage. Variables for healthcare resource use (number of GP and outpatient appointments in the previous 6 months) were excluded given the risk of poor recall and greater amounts of missing data; travel time to hospital was also excluded since it was not relevant for all responders. The final, parsimonious model was mutually adjusted for age, sex, marital status, home ownership, number of LTCs, number of prescribed regular medications, health literacy, and financial difficulty.

For the single-item measure, the sensitivity, specificity, positive predictive value, and negative predictive value at each integer number-line cut-off were calculated. ‘Correct’ identification of high treatment burden was determined relative to the MTBQ as a reference standard. A receiver operating characteristic curve was plotted, and area under the curve computed to evaluate the ability of the single-item measure to discriminate between high and non-high treatment burden.

## RESULTS

**Figure 1.**
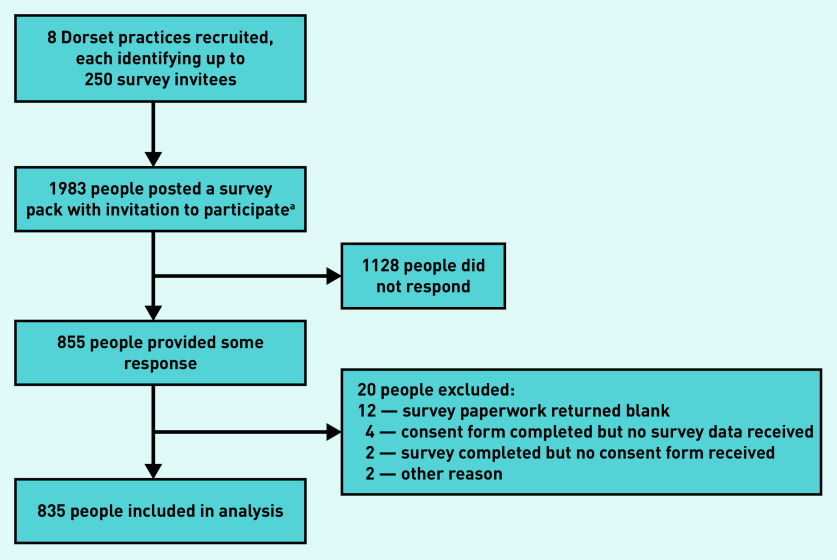
***Survey flowchart: recruitment, invitation, and response.*** *^a^****Seven practices invited 250 people; one practice invited 233 people.***

Eight practices were recruited, which exhibited heterogeneity in geographic location (including rurality/urbanity), deprivation level, and list size (see Supplementary Table S1 for details, which also presents practice demographics relative to national data). Manual exclusion of potentially eligible patients most commonly occurred at practice discretion (data from seven sites). In total, practices posted out 1983 surveys; 835 usable responses were received (response rate 42.1%), the vast majority by post (*n* = 808, 96.8%) (Figure 1).

With the exception of the questions relating to healthcare resource use, there were minimal missing data. A data entry error rate of <0.1% was estimated, which was deemed acceptably low. Age and sex distributions of the responder sample closely matched those of the invited sample (see Supplementary Table S2 for details). Responder characteristics are shown in Table 1. The mean age of responders was 75 years, 54.6% (*n* = 453) were females, and 99.2% (*n* = 822) were of white ethnicity. Responders were mostly retired (*n* = 716, 86.6%); many were married/in a civil partnership (*n* = 520, 63.0%); and most owned their own home (*n* = 654, 79.3%).

**Table 1.** Characteristics of survey responders (all data self-reported)

**Variable^a^**		**Number**	**% (of** ***N* for variable)**
**Age** in years, mean (SD)		75 (8.6)	

**Age category** in years *N* = 827 Missing *n* = 8 (1.0%)	55–59	40	4.8
	60–64	58	7.0
	65–69	125	15.1
	70–74	175	21.2
	75–79	173	20.9
	80–84	136	16.4
	85–89	83	10.0
	90–94	34	4.1
	≥95	3	0.4

**Sex** *N* = 829 Missing *n* = 6 (0.7%)	Male	376	45.4
	Female	453	54.6

**Ethnicity** *N* = 829 Missing *n* = 6 (0.7%)	White	822	99.2
	Other than white^b^	7	0.8

**Marital status** *N* = 826 Missing *n* = 9 (1.1%)	Married or in a partnership	520	63.0
	Widowed	180	21.8
	Divorced or dissolved partnership	94	11.4
	Single^c^	32	3.9

**Living situation** *N* = 827 Missing *n* = 8 (1.0%)	Cohabiting^d^	565	68.3
	Lives alone	262	31.7

**Home ownership** *N* = 825 Missing *n* = 10 (1.2%)	Home owner^e^	654	79.3
	Non-home owner	171	20.7

**Employment status** *N* = 827 Missing *n* = 8 (1.0%)	Retired	716	86.6
	Employed (full/part time)	67	8.1
	Unemployed	22	2.7
	Other	22	2.7

**Smoking status** *N* = 826 Missing *n* = 9 (1.1%)	Current smoker	44	5.3
	Ex-smoker	440	53.3
	Never smoked	342	41.4

**Number of long-term conditions^f^** *N* = 824 Missing *n* = 11 (1.3%)	0	7	0.8
	1	95	11.5
	2	223	27.1
	3	255	30.9
	4	138	16.7
	5	69	8.4
	≥6	37	4.5

**Medications prescribed** (number on repeat) *N* = 826 Missing *n* = 9 (1.1%)	0–3	130	15.7
	4–6	315	38.1
	7–9	204	24.7
	10–14	131	15.9
	≥15	46	5.6

**Treatment burden category** (Measure: MTBQ) *N* = 833 Missing *n* = 2 (0.2%)	High	150	18.0
	Medium	224	26.9
	Low	268	32.2
	None	191	22.9

**Health literacy** (frequency of needing reading help) (Measure: SILS) *N* = 830 Missing *n* = 5 (0.6%)	Never	554	66.7
	Rarely	137	16.5
	Sometimes	73	8.8
	Often	34	4.1
	Always	32	3.9

**Financial difficulty with** **health care** *N* = 828 Missing *n* = 7 (0.8%)	Not difficult or n/a	609	73.6
	A little	146	17.6
	Quite	44	5.3
	Very	24	2.9
	Extreme	5	0.6

**Travel time to hospital** *N* = 760 Missing or n/a, *n* = 75 (9.0%)	≤1 hour	707	93.0
	>1 hour	53	7.0

**Travel time to GP** *N* = 826 Missing *n* = 9 (1.1%)	≤10 minutes	505	61.1
	>10 minutes	321	38.9

**Number of outpatient** **appointments in last 6 months** *N* = 761 Missing *n* = 74 (8.9%)	0–2	605	79.5
	≥3	156	20.5

**Number of GP appointments** **in last 6 months** *N* = 724 Missing *n* = 111 (13.3%)	0–2	466	64.4
	≥3	258	35.6

a Missing percentages are based on overall survey responder denominator of 835.

b ’Other than white’ ethnicity comprises categories of ‘Mixed/multiple ethnic groups’, ‘Asian/Asian British’, ‘Black/African/Caribbean/black British’, or ‘Other ethnic group’, combined.

c Single means never married or in a civil partnership.

d Cohabiting means living with any of spouse/partner, child(ren), or other person.

e Home owner means owning or jointly owning one’s current home, including having a mortgage.

f For number of long-term conditions (LTCs): if responders had not ticked any LTC survey tick-box and not provided LTC freetext in the survey, LTC data are considered missing; if responders had not ticked any LTC tick-box but had provided some freetext (that did not itself indicate a survey-specified LTC), their LTC count is considered 0. MTBQ = Multimorbidity Treatment Burden Questionnaire. n/a = not applicable. p’ship = civil partnership. SILS = Single Item Literacy Screener.

A substantial minority (*n* = 325, 39.4%) self-reported fewer than three survey-specified LTCs, despite the presence of three or more such conditions in the GP record being an inclusion criterion (Table 1). The most frequently self-reported LTCs were hypertension (*n* = 531, 64.4%), osteoarthritis (*n* = 331, 40.2%), and type 2 diabetes (*n* = 248, 30.1%) (see Supplementary Table S3 for details). Polypharmacy was common, with 46.1% of responders (*n* = 381) prescribed seven or more medications (Table 1).

Some financial difficulty with health care (level of difficulty described as ‘a little’, ‘quite’, ‘very’, or ‘extreme’) was reported by 26.4% of responders (*n* = 219), and limited health literacy was reported by 16.7% (*n* = 139). The number of GP appointments was recalled as being three or more over the previous 6 months by 35.6% of responders (*n* = 258), and the number of hospital outpatient appointments as three or more over the same time period by 20.5% of responders (*n* = 156). For both these types of healthcare resource, responders being unable to remember/provide the number of appointments attended (despite indicating at least one attendance) was the principal reason for missing data.

**Figure 2.**
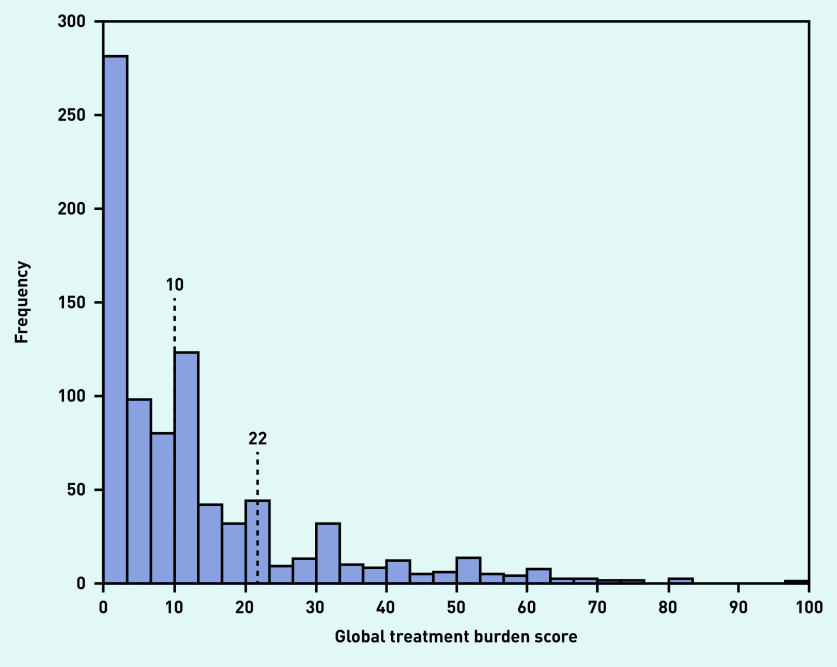
***Distribution of treatment burden (MTBQ global score) among survey responders.****^a^* *^a^* ***Global score 0 indicates no treatment burden; global score cut-offs of*** ≥***10 and*** ≥***22 (out of 100), indicated by the vertical dashed lines, facilitate categorisation of responders into those with ‘low’, ‘medium’, or ‘high’ treatment burden. MTBQ = Multimorbidity Treatment Burden Questionnaire.***

Distribution of global score for the 833 MTBQ responders is shown in Figure 2. ‘High’ treatment burden was reported by 18.0% (*n* = 150), ‘medium’ by 26.9% (*n* = 224), and ‘low’ by 32.2% (*n* = 268); no burden was reported by 22.9% (*n* = 191) (Table 1). Treatment burden domains in which responders most commonly reported ‘some difficulty’ (MTBQ item score >0) were: ‘making recommended lifestyle changes (such as diet/exercise)’ with 47.0% (*n* = 390) reporting difficulty; and ‘arranging appointments with health professionals’ (39.4%, *n* = 327) (see Supplementary Table 4 for details).

### Associations with high treatment burden

On univariable analysis, high treatment burden was associated with younger age; female sex; non-home-ownership; greater number of LTCs; more prescribed regular medications; limited health literacy; financial difficulty with health care; longer travel time to hospital; and more outpatient appointments, and GP appointments, in the previous 6 months.

The final multivariable model was mutually adjusted for age, sex, marital status, home ownership, number of LTCs, number of prescribed regular medications, health literacy, and financial difficulty. Strong independent associations (at *P*<0.001) were observed for both limited health literacy (odds ratio [OR] 3.64, 95% CI = 2.24 to 5.92) and financial difficulty (OR 3.94, 95% CI = 2.56 to 6.07), both being dichotomised variables. Independent associations were also seen for greater number of LTCs (overall *P* = 0.008 with ORs reaching significance at five or more LTCs, for example, OR 2.98 with 95% CI = 1.13 to 7.84 for 5 LTCs versus ≤1), and more prescribed regular medications (*P* = 0.04, OR 1.62, 95% CI = 1.03 to 2.55, for ≥7 medications versus <7) (Table 2).

**Table 2.** Univariable and multivariable associations with high treatment burden (versus non-high treatment burden)

**Characteristic^a^**		**Univariable**			**Multivariable^b^**		

		**OR**	**95% CI**	***P*-value**	**OR**	**95% CI**	***P*-value**
**Age category** in years (vs. 55–64)	65–74	0.60	0.36 to 1.01	**0.010**	0.82	0.44 to 1.56	0.268
	75–84	0.39	0.23 to 0.68		0.55	0.28 to 1.07	
	≥85	0.56	0.30 to 1.06		0.64	0.27 to 1.48	

**Sex** (vs. male)	Female	1.45	1.01 to 2.08	**0.046**	1.40	0.91 to 2.18	0.130

**Marital status** (vs. married)^c^	Single	1.52	0.64 to 3.63	0.348	1.32	0.49 to 3.57	0.579
	Divorced^d^	1.86	1.10 to 3.13	**0.020**	1.66	0.88 to 3.11	0.117
	Widowed	1.45	0.94 to 2.23	0.090	1.37	0.76 to 2.47	0.298

**Living situation** (vs. cohabiting)	Lives alone	1.15	0.79 to 1.68	0.461	—		

**Home ownership** (vs. home owner)	Non-home owner	2.32	1.56 to 3.43	<**0.001**	1.34	0.82 to 2.19	0.239

**Employment status** (vs. employed)	Unemployed	1.98	0.70 to 5.61	0.198	—		
	Retired	0.71	0.39 to 1.31	0.272			
	Other	0.77	0.23 to 2.63	0.677			

**Smoking** (vs. never smoked)	Ex-smoker	0.88	0.61 to 1.26	0.478	—		
	Current smoker	1.25	0.59 to 2.67	0.558			

**Number of long-term conditions** (vs. 0 or 1 condition)	2	1.25	0.56 to 2.79	<**0.001**	1.06	0.44 to 2.54	**0.008**
	3	2.41	1.13 to 5.11		1.91	0.83 to 4.39	
	4	2.42	1.08 to 5.42		1.21	0.50 to 3.00	
	5	6.25	2.70 to 14.47		2.98	1.13 to 7.84	
	≥6	8.78	3.43 to 22.52		4.38	1.42 to 13.53	

**Medications prescribed** (number on repeat) (vs.<7 medications)	≥7	2.66	1.84 to 3.86	<**0.001**	1.62	1.03 to 2.55	**0.038**

**Health literacy** (vs. not limited)	Limited	4.92	3.29 to 7.36	<**0.001**	3.64	2.24 to 5.92	<**0.001**

**Financial difficulty with health care** (vs. not difficult or n/a)	Some difficulty	5.18	3.55 to 7.54	<**0.001**	3.94	2.56 to 6.07	<**0.001**

**Travel time to hospital** (vs. ≤1 hour)	>1 hour	2.24	1.22 to 4.11	**0.009**	—		

**Travel time to GP** (vs. ≤10 minutes)	>10 minutes	1.31	0.91 to 1.89	0.140	—		

**Outpatient appointments in the last 6 months** (vs. 0–2)	≥3	2.57	1.71 to 3.86	<**0.001**	—		

**GP appointments in the last 6 months** (vs. 0–2)	≥3	3.14	2.12 to 4.64	<**0.001**	—		

a All characteristics are self-reported.

b Adjusting for age, sex, marital status, home ownership, number of long-term conditions, number of prescribed regular medications, health literacy, and financial difficulty.

c Married is used to refer to the category ‘Married or in a civil partnership’.

d Divorced is used to refer to the category ‘Divorced or dissolved civil partnership’. CI = confidence interval. n/a = not applicable. OR = odds ratio (for high treatment burden).

### Single-item treatment burden measure

**Figure 3.**
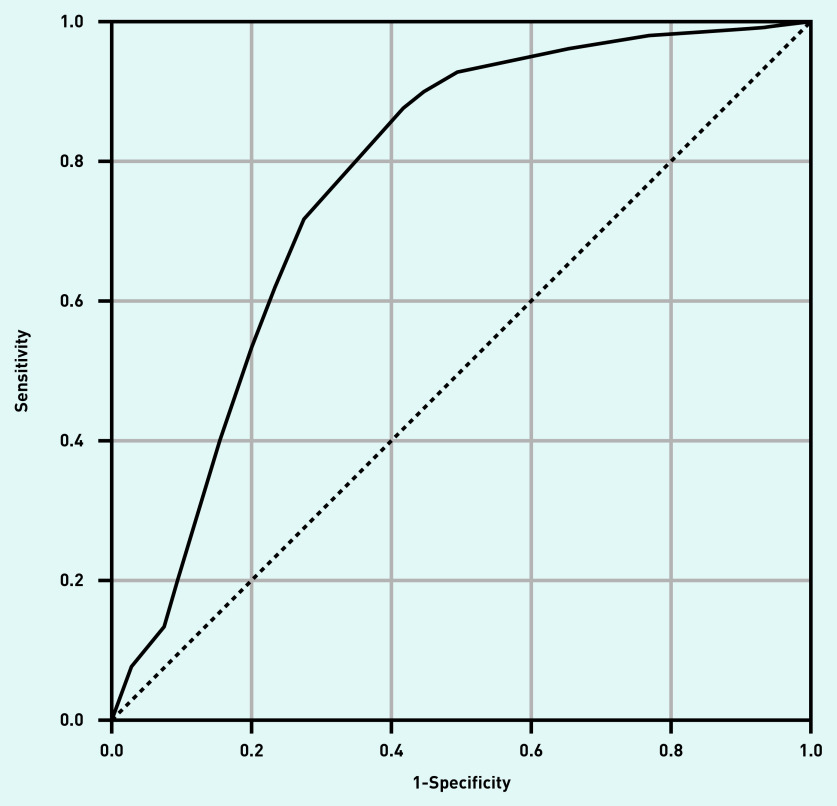
***Receiver operating characteristic curve for the exploratory single-item treatment burden measure.***

Setting a threshold of ≥5 for high treatment burden on the single-item measure (optimising its performance) yielded a sensitivity of 89%, specificity of 58%, positive predictive value of 31%, and negative predictive value of 96%. See Supplementary Table 5 for details of the underlying data for the 826 responders. Figure 3 displays the receiver operating characteristic curve; the area under the curve was 0.77 (95% CI = 0.73 to 0.81).

## DISCUSSION

### Summary

This cross-sectional study of older adults with multimorbidity identified that high self-reported treatment burden was reported by almost one-fifth of participants. Making recommended lifestyle changes and arranging appointments with health professionals frequently contributed to the burden. High treatment burden was strongly associated with limited health literacy and reported financial difficulty with health care; a greater number of LTCs and more prescribed regular medications were also independently associated. These findings imply that more vulnerable patients, with less capacity to manage, are at particular risk of being overburdened by healthcare demands. A novel single-item measure of treatment burden was explored, but performed moderately: further development of such a measure is required.

### Strengths and limitations

Strengths of this study include the number of responders considerably exceeding the minimum target sample size, generally little missing data, and virtually no non-response bias in age or sex. Regarding socioeconomic status, the proportion of homeowners, at 79%, is comparable to the 77% observed in those aged ≥55 years nationally (English Housing Survey 2018 to 2019 data),^29^ and the predominantly postal response supports generalisability beyond just those responders who are computer literate. The survey captured numerous characteristics including those indicative of patient capacity. Investigation of a single-item treatment burden measure was novel to the authors’ knowledge.

However, there are important limitations. Given the cross-sectional design, the study’s findings represent association, not causation. As is often seen with postal surveys, the absolute response rate was low (42%); while low response rate is not synonymous with non-response bias,^30^ characteristics of non-responders could not be assessed beyond age and sex. The proportion of patients with high treatment burden may be an underestimate if those experiencing the highest levels of burden were, as a consequence, less likely to respond. If non-responders also had lower levels of health literacy (which is plausible), the positive association identified between limited health literacy and high treatment burden might be underestimated. Socioeconomic status was captured only via the proxy measure of home ownership: a limitation, although home ownership has been shown to have some value as a marker of socioeconomic status, particularly among older people in Britain.^31^ Unaccounted confounding may also be occurring, for example, through the potential influence of carer burden or functional incapacity among responders; importantly, individual LTC severity was also not measured. The Single Item Literacy Screener captured patient-perceived reading ability: a central component of, but not wholly characterising, the multifaceted concept of health literacy.^32^ Despite home ownership levels of responders being comparable to those in England, caution is required before generalising findings from this Dorset study to other areas, given the below-national average deprivation profile of participating practices and predominantly white ethnicity of the sample.

Almost 40% of responders self-reported fewer than three LTCs, at variance with their GP record. This could reflect limited recall or lack of awareness if diagnoses were not discussed by clinicians; this is well recognised in some conditions such as chronic kidney disease.^33^ Imprecision associated with electronic patient record system coding may also be a contributory factor.^34^ The association of treatment burden with LTC count should therefore be interpreted with caution, but is highly plausible.

The single-item treatment burden measure is acknowledged as entirely exploratory and was not subject to formal development. The item’s discriminatory ability, based on the area under the curve, was fair; specificity and positive predictive value were low, but sensitivity and negative predictive value were high, for a number-line threshold ≥5, suggesting some utility in ‘ruling out’ high treatment burden, rather than in screening for high burden *per se*. It is possible that the position of the single-item relative to other questions (the item was located part-way through the survey) influenced responses to the item. Further development is clearly needed; current item formulation may provide a starting point for iterative work with patient groups.

### Comparison with existing literature

Distribution of treatment burden was comparable to that observed during MTBQ validation in the 3D study.^21^^,^^35^ 3D exhibited a greater proportion with high burden (27% versus 18%), potentially explained by a likely more multimorbid sample (from grouping of similar LTCs within selection criteria, and inclusion of severe mental health conditions), and inclusion of younger participants (≥18 years versus ≥55 years) who tended to report greater burden. An inverse age–burden relationship was also observed among European cohorts with chronic conditions^36^^,^^37^ (and here, although non-significant after adjustment); this could perhaps result from balancing employment demands or caring responsibilities with LTC management, or denote differing healthcare-related expectations with age. This study did not identify an independent association with female sex that has been noted previously.^21^ However, as here, during MTBQ development an association between the number of LTCs and high treatment burden was observed;^21^ such a relationship is consistent with presumed connections between multimorbidity and treatment burden.

Making recommended lifestyle changes and arranging appointments with health professionals were the domains most commonly generating ‘some difficulty’ — likewise observed during MTBQ development.^21^ This consistent finding could direct burden-reduction initiatives.

Correlation between high treatment burden and markers of workload (more pharmacological treatments, more LTCs) was observed in a Swiss cohort with multimorbidity; however, this was for GP-assessed burden compared with patient-perceived burden.^37^ Qualitative work indicates medications can contribute to burden (for example, by interfering with activities, or requiring coordination),^38^ consistent with the association identified in this study with more prescribed regular medications (independent of number of LTCs).

Non-home-ownership was associated with high treatment burden on univariable analysis; in the multimorbid Swiss cohort, socioeconomic deprivation was independently associated with high burden.^37^ A Danish study of 2111 people with cardiovascular disease and multimorbidity also found greater treatment burden among those with difficulty understanding health information^39^ — consistent with the findings of the current study, and suggesting that enhancing health literacy (and thereby capacity) might mitigate burden.

Associations with limited health literacy and financial difficulty accord with Shippee *et al’s*^13^ ‘cumulative complexity model’: lower levels of capacity to manage workload relating to greater burden. A mixed-methods study in the US, however, found that capacity indicators were seldom documented in medical records,^40^ implying under-recognition of capacity issues by clinicians: a potential barrier to prospective capacity-enhancing interventions.

Patients with multimorbidity report various strategies to lessen perceptions of burden, for example, maintaining a positive attitude or normalising self-care, and, notably, drawing on positive aspects of health care.^41^ Indeed, provider communication and interpersonal skills (‘relational quality’) have been correlated with lower treatment burden.^42^ Qualitative work with stroke professionals similarly identified communication and coordination, as part of individualised care, as vital to reducing treatment burden.^43^ This links importantly to the current study because of the finding that limited health literacy was associated with high treatment burden.

### Implications for research and practice

Almost one-fifth of older adults with multimorbidity in this study reported high treatment burden, underlining the importance of recognising, and seeking to minimise, avoidable burden. While this study has focused on those with three or more documented LTCs, there remains the potential for patients with fewer conditions to also experience treatment burden. Further work is needed to investigate changes in treatment burden over time, and the impact of workload and capacity on perceived burden, adherence, and health outcomes. Research evaluating the impact of interventions that might reduce workload or enhance capacity would also be beneficial. As of 2020, such enquiry may be facilitated by innovations stimulated by the COVID-19 pandemic, including the introduction of potentially more sustainable models of healthcare delivery.^44^ Social prescribing,^45^ now increasingly available via primary care, might contribute to bolstering patient capacity, for example, by enhancing support networks or facilitating improvement in wellbeing. Principles of ‘minimally disruptive medicine’ will be key to reducing burden, including care coordination, development of clinical guidelines tailored to comorbidity, and prioritisation of care from the patient perspective.^16^ System-level healthcare solutions are required, not only those applicable at individual patient level.

The ability to assess treatment burden swiftly and accurately is nevertheless key to optimising care, hence a single-item measure could ultimately find utility in clinical settings. Given the moderate performance of the single-item treatment burden measure explored in this study, this item should not yet be adopted into practice: further development is recommended.

Clinicians should be alert to potentially overburdened patients. The study findings suggest that, in addition to those with a greater number of LTCs and more prescribed regular medications, patients with limited health literacy and fewer financial resources are at increased risk of high treatment burden. Such factors indicate increased vulnerability and reduced capacity to manage the work of looking after one’s health. GPs have a central role in incorporating these considerations into patient care for those with multimorbidity, to ensure that patients are not overburdened with healthcare demands.
